# Calculation of multipoint likelihoods using flanking marker data: a simulation study

**DOI:** 10.1186/1471-2156-6-S1-S44

**Published:** 2005-12-30

**Authors:** Andrew W George, LaVonne A Mangin, Christopher W Bartlett, Mark W Logue, Alberto M Segre, Veronica J Vieland

**Affiliations:** 1 Program in Public Health Genetics, College of Public Health, University of Iowa, Iowa City, USA; 2 Department of Computer Science, College of Liberal Arts and Sciences, University of Iowa, Iowa City, USA; 3 Department of Internal Medicine and Carver College of Medicine, University of Iowa, Iowa City, USA; 4 Department of Psychiatry, Roy L. and Lucille A. Carver College of Medicine, University of Iowa, Iowa City, USA

## Abstract

The calculation of multipoint likelihoods is computationally challenging, with the exact calculation of multipoint probabilities only possible on small pedigrees with many markers or large pedigrees with few markers. This paper explores the utility of calculating multipoint likelihoods using data on markers flanking a hypothesized position of the trait locus. The calculation of such likelihoods is often feasible, even on large pedigrees with missing data and complex structures. Performance characteristics of the flanking marker procedure are assessed through the calculation of multipoint heterogeneity LOD scores on data simulated for Genetic Analysis Workshop 14 (GAW14). Analysis is restricted to data on the Aipotu population on chromosomes 1, 3, and 4, where chromosomes 1 and 3 are known to contain disease loci. The flanking marker procedure performs well, even when missing data and genotyping errors are introduced.

## Background

The calculation of multipoint likelihoods on general pedigrees is computationally challenging. Factors influencing the complexity of multipoint calculations include family size, pedigree structure, marker number, and missing data. Efficient algorithms have been developed for handling large pedigrees with few markers [1] or small pedigrees with many markers [2], but calculating multipoint probabilities on large pedigrees with many markers is infeasible.

In this paper, the performance characteristics of CHROM-WALK, a computer program for calculating multipoint likelihoods on general pedigrees and many linked markers, is explored. Multipoint likelihoods are calculated using data observed only on markers flanking a hypothesized position of the trait locus. Calculating these three-point likelihoods is often feasible even on large complex pedigrees. Likelihood computations in CHROM-WALK are performed via VITESSE [3]. The speed and accuracy of CHROM-WALK are examined through a heterogeneity LOD (HLOD) score analysis of data simulated on the Aipotu population from Genetic Analysis Workshop (GAW) 14. CHROM-WALK has been developed to make the multilocus linkage analysis of data on large general pedigrees computationally feasible.

## Methods

### CHROM-WALK

The CHROM-WALK computer program uses VITESSE to perform likelihood calculations needed to calculate three-point likelihoods across a chromosome on general pedigrees. Multipoint likelihoods are calculated on data on markers flanking a hypothesized position of the trait locus. Results are reported as either homogeneity LOD scores or HLOD scores at pre-specified positions of the trait locus. Functionally, CHROM-WALK is similar to GENEHUNTER [4]. Only a single locus file and pedigree file in linkage format are required as input files. Command line arguments are used to specify the distance (in cM) between hypothesized positions, the distance beyond the linkage map (if any) to compute likelihoods, and whether homogeneity LOD scores or HLOD scores are to be reported.

### Simulated data

In this study, data generated on the GAW14 Aipotu population were chosen for analysis. This data consisted of 100 nuclear families, ranging in size from 2 to 10 siblings. For computational expedience, the other three GAW 14 populations were not analyzed. Marker and trait data were observed on each individual. The trait is dichotomous where an individual is either affected or unaffected for the disease. Microsatellite marker data on chromosomes 1, 3, and 4, containing 41, 42, and 44 linked markers, respectively, are selected for analysis. Inter-marker distances, on average, are 7.5 cM. Chromosome 1 contained a disease locus between the 23^rd ^and 24^th ^marker locus. Chromosome 3 contained a disease locus between the 41^st ^and 42^nd ^marker locus. Chromosome 4 is unlinked to disease causing loci. There are 100 replicates of data.

### Linkage detection and mapping

The accuracy of CHROM-WALK for detecting and localizing trait loci was examined through the analysis of simulated family data. A dominant trait model with incomplete penetrance (0.05, 0.95, 0.95) and a disease allele frequency of 0.01 was assumed. Here, three marker sets formed from the original GAW14 simulated data were considered: four linked markers closest to the disease locus (Mset4), 16 linked markers closest to the disease locus (Mset16), and all markers on a chromosome (MsetAll). Because chromosome 4 is unlinked to a disease locus, markers were selected from the beginning of the marker map. HLOD scores are calculated every 1 cM. Three-point HLOD scores are compared to multipoint HLOD scores calculated via GENEHUNTER. Multipoint scores on each data set are only calculated on markers available in that data set. Hence, the impact of increasing the number of markers incorporated into the multipoint calculation can be examined.

### Missing data and genotyping errors

Multipoint calculations on pedigrees are affected by missing data and genotyping error. To explore the utility of CHROM-WALK given imperfect data, missing data and Mendelian consistent genotyping errors were introduced. The marker phenotype at a locus for an individual was randomly removed with probability 0.01. Mendelian consistent genotyping errors were created, with probability 0.005, by randomly permuting with equal probability the transmitted allele from one of the parents. Note that this error model is simplistic since it cannot produce genotyping errors in the parents and does not make distinctions between types of genotyping errors, which are all equally likely in the present study. The assumed probability of Mendelian consistent errors was consistent with an overall (pedigree consistent and inconsistent) genotyping error rate of 1% [5]. The levels of missing data and genotyping error were realistic compared to real data.

## Results

### Linkage detection and mapping

To examine the accuracy of calculating multipoint likelihoods using flanking markers, mean HLOD scores averaged over the 100 replicates are calculated at each hypothesized position of the trait locus for chromosomes 1, 3, and 4 for marker sets Mset4, Mset16, and MsetAll. There is close agreement between the CHROM-WALK and GENEHUNTER HLOD scores across hypothesized positions of the trait locus. The mean scores at the known location of the trait locus for chromosomes 1 and 3 and at an arbitrary chromosomal position for chromosome 4 are reported in Table 1. Given the associated standard errors, the difference between the CHROM-WALK and GENEHUNTER HLOD scores is insignificant. Also, there is little change in mean GENEHUNTER HLOD scores across marker sets.

**Figure 1:**
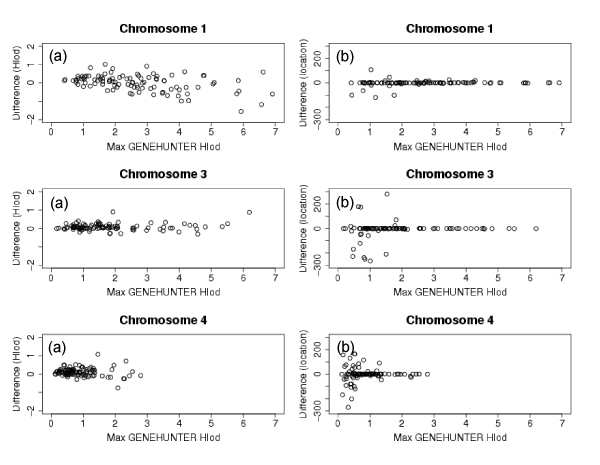
**Differences in peak HLOD scores and differences in inferred locations of trait locus. **(a) Difference between the GENEHUNTER peak HLOD score and the CHROM-WALK peak HLOD score against the GENEHUNTER peak HLOD score for analyses of data on chromosomes 1, 3, and 4. (b) Difference between the chromosomal locations of the peak HLOD scores against the GENEHUNTER peak HLOD score for analyses of data on chromosomes 1, 3, and 4. Each point represents results from the analysis of a single data replicate for marker set MsetAll.

To examine the utility of CHROM-WALK for detecting and localizing trait loci, differences in the peak GENEHUNTER and CHROM-WALK HLOD scores and differences in the chromosomal location of the peaks are investigated. Figure 1a plots the difference in peak HLOD scores on the vertical axis against the peak GENEHUNTER HLOD on the horizontal axis for the analysis of data on MsetAll. That is, each point represents the difference in CHROM-WALK and GENEHUNTER linkage results from the analysis of a single replicate. In Figure 1a, there are a cluster of points around a horizontal line intersecting 0 on the vertical axis indicating near perfect results. Points off of the horizontal line indicate differences in peak scores. However, it is reassuring that the largest differences in peak scores occur when the peak GENEHUNTER score is also large. Using HLOD scores calculated on flanking markers for detection does not result in conclusions that are different to analyzing data on all available markers jointly.

Figure 1b plots the difference in the chromosomal location of the peaks on the vertical axis against the peak GENEHUNTER HLOD on the horizontal axis. Again, there is a clustering of points around a horizontal line intersecting 0 on the vertical axis, indicating close agreement between the localization of the trait using flanking markers and all available markers. It is also reassuring that the largest differences in locations occur for small peak GENEHUNTER scores. When the peak GENEHUNTER score is small, there is little information in the data for detecting linkage. Using HLOD scores calculated on flanking markers for localization does not result in conclusions that are different to analyzing data on all available markers jointly.

### Missing data and genotyping errors

**Figure 2:**
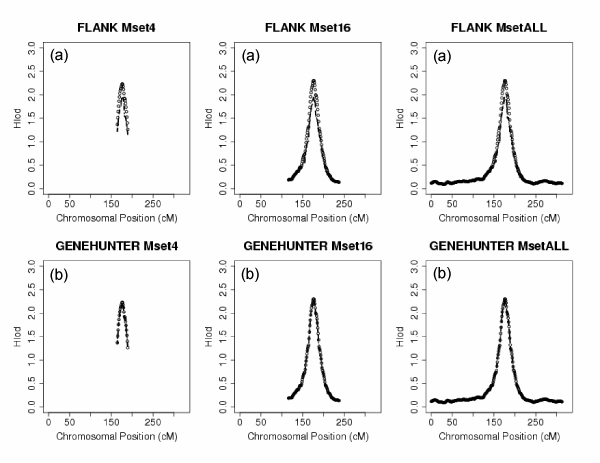
**Comparison of mean HLOD scores calculated using CHROM-WALK and GENEHUNTER on chromosome 1 data. **Mean HLOD scores, calculated using CHROM-WALK (a) and GENEHUNTER (b) on chromosome 1 data. Circles represent GENEHUNTER HLOD scores, calculated on data with no missing data. The dashed line represents the HLOD score curve calculated on data with 1% missing data and 0.5% Mendelian consistent genotyping error.

Results are reported for the analysis of chromosome 1 in which both missing data and genotyping errors were randomly introduced. Results from the analysis of chromosome 3 and 4 data and data where either missing data or genotyping errors were introduced are not reported but are consistent with the analysis presented here. Mean CHROM-WALK and GENEHUNTER HLOD scores at hypothesized positions of the trait locus for data on chromosome 1 for the three markers sets (Mset4, Mset16, MsetALL) are plotted in Figure 2. Means are calculated from the analysis of the 100 data replicates on the Aipotu population. For comparison, each plot also contains the mean GENEHUNTER HLOD scores on data without errors or missing information (circles).

From Figure 2, the mean HLOD scores calculated using CHROM-WALK and GENEHUNTER show that the scores are quite robust to imperfect data. The mean CHROM-WALK HLODs (the dashes in Figure 2a) are slightly lower but there is still clear evidence of linkage. The mean GENEHUNTER HLODs calculated on the imperfect data (the dashes in Figure 2b) are almost identical to the GENEHUNTER HLODs with perfect data (the circles in Figure 2b). Furthermore, it is reassuring that the CHROM-WALK HLODs are similar to the GENEHUNTER HLODs across marker subsets, despite GENEHUNTER requiring an order of magnitude longer run times for the analysis of most replicates.

## Conclusion

In this paper, the calculation of multipoint likelihoods using a new computer program CHROM-WALK is assessed through the calculation of HLOD scores on simulated data. By only considering data observed on flanking markers, the computational complexity of multipoint calculations are greatly reduced. For data simulated on nuclear families, there is little loss in accuracy using the proposed approximation procedure. Furthermore, CHROM-WALK produced multipoint results, on average, an order of magnitude faster than GENEHUNTER. Further exploration is warranted for extended families, differing amounts and patterns of missing data, differing amounts of genotyping error, and changes in marker informativeness.

## Abbreviations

GAW14: Genetic Analysis Workshop 14

HLOD: Heterogeneity LOD score

## Authors' contributions

AWG performed the analyses and wrote paper. LAM, AMS, and VJV developed CHROM-WALK software. MWL and CWB involved in the design of the simulation study, reporting of results and refining of the manuscript.

## Figures and Tables

**Table 1:** Comparison of CHROM-WALK and GENEHUNTER HLOD scores for different marker sets

Chr	Chr 1 Pos	Mset4		Mset16		MsetAll	
				
		HLOD_FL_	HLOD_GH_	HLOD_FL_	HLOD_GH_	HLOD_FL_	HLOD_GH_
1	175 cM	1.96 (0.15)	2.23 (0.15)	1.95 (0.15)	2.30 (0.16)	1.94 (0.15)	2.30 (0.16)
3	312 cM	1.57 (0.14)	1.57 (0.14)	1.57 (0.14)	1.57 (0.14)	1.57 (0.14)	1.57 (0.14)
4	20 cM	0.06 (0.02)	0.06 (0.02)	0.06 (0.02)	0.06 (0.02)	0.06 (0.02)	0.06 (0.02)

Mean GENEHUNTER HLOD (HLOD_GH_) scores and mean CHROM-WALK HLOD (HLOD_FL_) scores, averaged over 100 simulated replicates of the Aipotu population, at a location between markers flanking the trait locus for chromosomes 1 and 3. For chromosome 4, mean HLOD scores are reported at an arbitrary chromosomal position, common to all three marker sets Mset4, Mset16, and MsetAll. GENEHUNTER multipoint scores are calculated on markers available within each data set. CHROM-WALK multipoint scores are calculated only on flanking markers jointly. Standard errors of the means are given in parentheses.
